# Effect of Dietary Glycemic Index on β-Cell Function in Prediabetes: A Randomized Controlled Feeding Study

**DOI:** 10.3390/nu14040887

**Published:** 2022-02-19

**Authors:** Alexander T. Sipe, Marian L. Neuhouser, Kara L. Breymeyer, Kristina M. Utzschneider

**Affiliations:** 1 Creighton University School of Medicine, Omaha, NE 68124, USA; alexandersipe@creighton.edu; 2 Public Health Sciences Division, Fred Hutchinson Cancer Research Center, Seattle, WA 98109, USA; mneuhous@fredhutch.org (M.L.N.); karabrey@gmail.com (K.L.B.); 3 Research and Development, Department of Medicine, VA Puget Sound Health Care System, Seattle, WA 98108, USA; 4 Division of Metabolism, Endocrinology and Nutrition, Department of Medicine, University of Washington, Seattle, WA 98195, USA

**Keywords:** glycemic index, prediabetes, insulin secretion, beta-cell function, pathophysiology, adults

## Abstract

The glycemic index (GI) reflects the relative ability of carbohydrates to raise blood glucose. We utilized a controlled feeding study to assess the impact of the dietary GI on β-cell function in adults with prediabetes (17F/18M, mean ± SEM: BMI 32.44 ± 0.94 kg/m^2^, age 54.2 ± 1.57 years). Following a 2 week Control diet (GI = 55–58), participants were randomized to either a 4 week low GI (LGI: GI < 35, n = 17) or high GI (HGI: GI > 70, n = 18) diet (55% of energy from carbohydrate/30% fat/15% protein). The data from 4 h meal tolerance tests (MTTs) underwent mathematical modeling to assess insulin sensitivity, insulin secretion and β-cell function. Glucose concentrations during the MTT decreased on the LGI diet (*p* < 0.001) and trended to increase on the HGI diet (*p* = 0.14; LGI vs. HGI *p* < 0.001), with parallel changes in insulin and C-peptide concentrations. Total insulin secretion, adjusted for glucose and insulin sensitivity, increased on the LGI diet (*p* = 0.002), and trended lower on the HGI diet (*p* = 0.10; LGI vs. HGI *p* = 0.001). There was no significant diet effect on insulin sensitivity or other measures of β-cell function. Total insulin clearance increased on the LGI diet (*p* = 0.01; LGI vs. HGI *p* < 0.001). We conclude that short-term consumption of an LGI diet reduced glucose exposure and insulin secretion but had no impact on measures of β-cell function.

## 1. Introduction

According to the Centers for Disease Control and Prevention, 45% of adults in the United States have type 2 diabetes or prediabetes [1]. Hyperglycemia results from a mismatch between the insulin secretion from the pancreatic β-cells and the ability of tissues to take up glucose in response to insulin. Although type 2 diabetes is characterized by both insulin resistance and a β-cell defect, β-cell dysfunction is considered the critical feature in the development of type 2 diabetes [2] and is present well before the diagnosis of type 2 diabetes [3,4]. Reversing β-cell dysfunction in those at risk for developing diabetes may be possible if there is early intervention [5].

Lifestyle approaches are recommended to reduce the risk of progression to diabetes and include weight loss, increased physical activity, and diets reduced in fat and calories [6]. Controlling the glycemic index (GI) of the diet has also been used as an approach to control the glucose response to carbohydrates [7]. The GI of a food is a value assigned to it that details how quickly and to what extent the food increases blood glucose [8,9]. For example, white bread has a high GI (GI 73), while pumpernickel bread has a low GI (GI 46). A meta-analysis of observational studies found that the GI of the diet was positively associated with an elevated risk of incident type 2 diabetes (RR 1.40, 95% CI: 1.23, 1.59) [10] and thus, may be a modifiable factor that could reduce the risk of diabetes.

Two separate meta-analyses of randomized controlled studies in individuals with diabetes found that low GI diets had small but meaningful effects on glycemic control [7,11]. In a randomized trial of adults with type 2 diabetes, dietary advice to follow a low GI diet did not impact insulin sensitivity, but the disposition index, a measure of β-cell function, improved after 12 months [12]. Evidence also exists that the GI may be important in the prevention of type 2 diabetes. In prospective observational studies, dietary GI was independently associated with the risk of developing type 2 diabetes [13,14]. Similar to the study in type 2 diabetes, adults with impaired glucose tolerance (IGT), randomized to dietary advice to follow a low GI diet, had no change in insulin sensitivity, but an improvement in the disposition index [15]. Few controlled feeding studies have examined the effect of dietary GI on insulin sensitivity [16,17] or β-cell function [16].

Given the paucity of well controlled feeding studies, we undertook a controlled dietary intervention to assess the impact of 4 weeks of a low GI vs. a high GI diet on insulin sensitivity and β-cell function. We specifically enrolled adults with prediabetes as they are known to have β-cell dysfunction and be at high risk of developing type 2 diabetes over time. The study protocol included a baseline 2 week Control diet, with a moderate GI, followed by 4 weeks on either a low GI (LGI) or a high GI (HGI) diet. We utilized modeling of glucose, insulin, and C-peptide during a mixed meal tolerance test (MTT) to ascertain in a physiologic setting, the effect of the GI on measures of insulin sensitivity and β-cell function. We hypothesized that the LGI diet would result in lower glucose concentrations, as well as lower insulin secretion and greater sensitivity of the β-cell to glucose compared to the HGI diet.

## 2. Materials and Methods

Study Design: The study was a randomized, parallel design, controlled feeding study in men and women with prediabetes. Subjects were randomly assigned to either an LGI or HGI diet. The data being presented here are from the 4 h MTT, which was performed at the end of the control and intervention diet periods, and do not include data from a third study arm that added the antioxidant N-acetyl-cysteine to a HGI diet. The results from the frequently sampled intravenous glucose tolerance test (IVGTT) and continuous glucose monitoring are previously published [18]. The activities for the controlled feeding portion of the study were conducted at the Fred Hutchinson Cancer Research Center; recruitment, screening and all other study procedures were completed at the VA Puget Sound outpatient Diabetes Research Group clinic in Seattle, WA. The study is registered under clinicaltrials.gov as NCT01386645.

Study Participants: Enrollment occurred from 2012–2017. Recruitment utilized general advertisements as well as targeted mailings to individuals identified by electronic medical record searches as being at risk for prediabetes or type 2 diabetes. All the interested participants were screened with a 75 g 2 h oral glucose tolerance test, fasting labs, and a review of their medical history and diet intolerances to determine eligibility. Eligibility criteria included: age 18–65 years old and impaired glucose tolerance (IGT: 2 h glucose 7.78–11.056 mmol/L (140–199 mg/dL)) and/or impaired fasting glucose (IFG: fasting plasma glucose 5.56–6.39 mmol/L (100–115 mg/dL) with 2 h glucose > 5.56 mmol/L (100 mg/dL)) on a screening oral glucose tolerance test (OGTT). Exclusion criteria included fasting glucose > 6.39 mmol/L (116 mg/dL), known diabetes, elevated liver enzymes (alanine aminotransferase > 1.5 times the upper limit of normal), anemia (hematocrit < 33%), creatinine > 1.5 mg/dL for men or > 1.3 mg/dL for women, food allergies or intolerances, current medication use that could affect insulin sensitivity or secretion, gastrointestinal issues, pregnancy, lactation, tobacco use or other serious medical conditions. Participants were randomized using a computer-generated randomization sequence stratified by sex. Diet assignment was not blinded.

Diet Intervention: Participants completed a baseline Control diet for 2 weeks that contained carbohydrates with a moderate GI and underwent baseline testing at completion of the Control diet period. The following day they started the LGI or HGI diets which they consumed for a total of 4 weeks before undergoing repeat testing. Details on the diets are provided elsewhere [18]. In brief, study diets were designed by the Fred Hutchinson Cancer Research Center Human Nutrition Lab (HNL), based on a previous study [19], to have stable macronutrient composition (55% of energy from carbohydrate/30% fat/15% protein). The average daily GI for each diet was designed to be 55–58 for the baseline Control diet, >70 for the HGI diet and <35 for the LGI diet. All the food was provided to study participants and caloric intake was adjusted throughout, based on biweekly weights, to maintain weight stability.

Meal Tolerance Test (MTT): An MTT was performed at the end of the Control diet and again at the end of the LGI or HGI diet periods. After at least a 12 h overnight fast, participants underwent a 4 h frequently sampled intravenous glucose tolerance test, followed by the 4 h MTT. The meal for the MTT was specifically designed to contain 75 g of carbohydrate and to mimic the GI of the diet being consumed at the time of the test (Control diet (mean GI = 52), LGI (GI = 29), HGI (GI = 86)) and contained a similar macronutrient composition (See Supplemental Table S1 for the MTT meal composition). For the MTT, samples were drawn 5 and 1 min prior, and 10, 20, 30, 60, 90, 120, 150, 180, 210 and 240 min after the start of the meal ingestion. The meal was consumed within 20 min. Blood samples were collected in tubes containing EDTA and placed immediately on ice, centrifuged at 4 °C for 15 min, and plasma aliquoted into cryovials that were frozen at −80 °C until assayed.

Assays: Plasma glucose was assayed using the glucose oxidase method. Plasma insulin was measured using an automated electrochemiluminescence immunoassay (Cobas e 601, Indianapolis, IN, USA). C-peptide was assayed using Tosoh reagents on a TOSOH 2000 autoanalyzer (TOSOH, Biosciences, Inc., South San Francisco, CA, USA).

Assessment of insulin sensitivity and β-cell function: Model parameters were estimated from the MTT glucose and C-peptide concentrations using C-peptide deconvolution [20], as previously described [21]. Using a mathematical model [21,22], β-cell glucose sensitivity (the slope relating insulin secretion rate (ISR) to glucose concentration) and ISR at a fixed reference glucose concentration (5 mmol/L: ISR@gref5) were determined. The integral of ISR during the whole test (total ISR) was calculated and adjusted for the glucose stimulus by dividing by the incremental area under the curve glucose (incAUCglu). The dose-response is modulated by a potentiation factor, which expresses relative potentiation or inhibition of the ISR; its excursion is quantified by the ratio between the time interval from the latter part of the test (220–240 min) relative to the early time period (0–20 min) (potentiation ratio). The ISR is also dependent on the rate of change of glucose determined by a single parameter termed rate sensitivity, which is related to early insulin release [23]. Oral glucose insulin sensitivity (OGIS) was estimated by the modeling of glucose and insulin data from the first 2 h of the MTT [24]. Modeling was performed using Matlab R2018b (Mathworks, Natick, MA, USA).

Calculated measures: The early insulin and C-peptide responses to glucose were calculated as the change in insulin or C-peptide divided by the change in glucose from 0 to 30 min. Incremental area under the curve (incAUC) insulin (ins), C-peptide (cp) and glucose (glu) responses were calculated using the trapezoidal rule and the incAUCins/incAUCglu and incAUCcp/incAUCglu computed. The Matsuda index of insulin sensitivity was calculated as 10,000/(SQRT(fasting glucose × fasting insulin) × (mean OGTT glucose × mean OGTT insulin)) [25].

Statistical Analysis: 18 participants were randomized to the LGI diet and 19 to the HGI diet. One participant on the LGI diet was suspected of having eaten before the second set of tests and the data deemed unreliable and one participant on the HGI diet was lost to follow-up (See Consort Diagram Supplemental Figure S1). Thus, 35 participants are included in this analysis. A sample size of 16 subjects/arm was determined for the parent study’s primary outcome of the disposition index from an IV glucose tolerance test [18].

Data that were not normally distributed are depicted as median (interquartile range (IQR)) and were natural log transformed for all statistical analyses. Baseline characteristics between the LGI and HGI arms were compared using ANOVA for continuous variables and chi-square for categorical variables. Paired *t*-test analysis evaluated for diet effect within each arm. To compare the effect of the LGI vs. the HGI intervention, generalized linear estimating equations (GEE) were run, with the outcome variable as the dependent variable, diet arm as a factor, and the baseline as a covariate. As age differed between the LGI and HGI arms, it was also included as a covariate in all GEE analyses. For GEE analysis of variables that are dependent on insulin sensitivity (total ISR, ISR@gref5, the early insulin and C-peptide responses, incAUCins/incAUCglu and incAUCcp/incAUCglu), OGIS from the second MTT was also included as a covariate. Glucose sensitivity from the modeling was the primary outcome. A *p* value < 0.05 was considered significant. Analyses were run using SPSS version 28.0.0.0 (IBM Corporation, Armonk, NY, USA).

## 3. Results

### 3.1. Baseline Characteristics

Overall, the LGI arm was, on average, older than the HGI arm (*p* = 0.01), but there were no significant differences in BMI, HbA1c, fasting glucose, 2 h glucose, race, or ethnicity. There was an equal distribution of those with IFG, IGT, or both IFG + IGT (Table 1). Compliance was based on the amount of carbohydrates planned vs. consumed, based on any food items returned, and was >99% overall. Weight decreased in both the LGI and HGI arms but did not differ between the intervention arms (mean change ± SEM: LGI −1.71 ± 0.66 kg, paired *t*-test *p* = 0.006; HGI 0.42 ± 0.24 kg, paired *t*-test *p* = 0.095; GEE: LGI vs. HGI *p* = 0.35).

### 3.2. Effect of Dietary Glycemic Index on MTT Glucose, Insulin and C-Peptide

**Figure 1:**
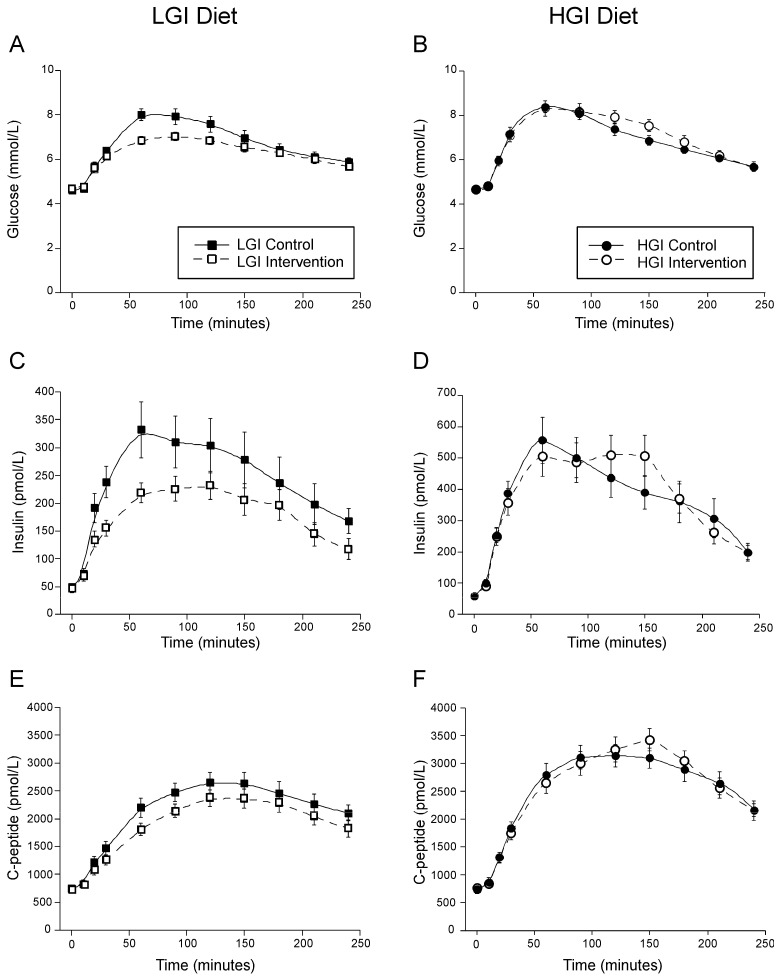
Glucose (**A**,**B**), insulin (**C**,**D**) and C-peptide (**E**,**F**) concentrations during the MTT. The LGI diet is depicted by squares and the HGI diet by circles. The Control diet is denoted by solid symbols and a solid line, and the intervention diet by open symbols and a dashed line. Mean ± SEM.

The glucose levels throughout the MTT reflected the intervention diet that the subjects were assigned. The LGI MTT resulted in lower concentrations of glucose (Figure 1A) and incAUCglu (Table 2), while the HGI MTT resulted in only slightly higher glucose concentrations in the middle of the MTT (Figure 1B), and a nonsignificant increase in the incAUCglu (Table 2), with significant between arm differences by GEE analysis. A similar pattern occurred in insulin and C-peptide concentrations across the MTT in response to the respective intervention diets (Figure 1C–F). The LGI diet resulted in a lower early insulin response and higher incAUC C-peptide response to glucose, but no change in the early C-peptide response or incAUC insulin response to glucose. The only significant change in these responses on the HGI diet was a decrease in the incAUC C-peptide response to glucose. When evaluated by GEE analysis, there was a significant diet effect only on the incAUC C-peptide response, with it increasing on the LGI diet (Table 2). All insulin and C-peptide responses were adjusted for insulin sensitivity.

### 3.3. Effect of Dietary Glycemic Index on Insulin Secretion

**Figure 2:**
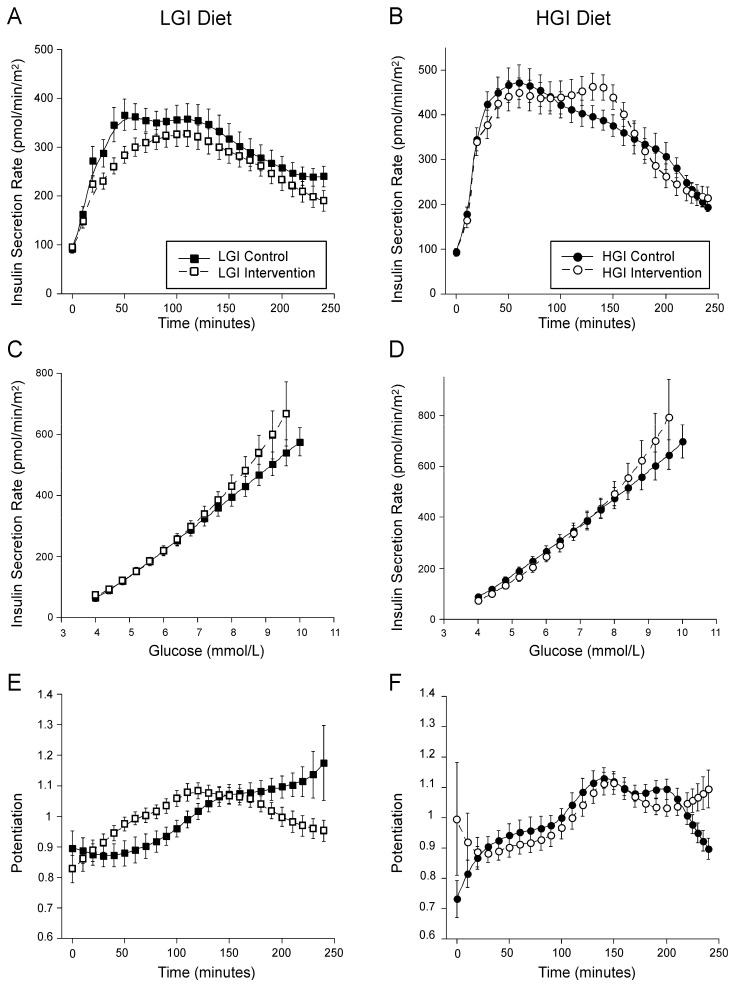
ISR vs. time (**A**,**B**), ISR vs. glucose dose response (**C**,**D**) and potentiation (**E**,**F**) from modeling of glucose and C-peptide concentrations during the MTT. The LGI diet is depicted by squares and the HGI diet by circles. The Control diet is denoted by solid symbols and a solid line and the intervention diet by open symbols and a dashed line. Mean ± SEM.

Insulin secretion at a reference glucose of 5 mmol/L glucose did not change with the LGI diet but decreased on the HGI diet (Table 2). Total insulin secretion rates were lower on the LGI diet but did not change on the HGI diet compared to the Control diet; there was a significant difference between the LGI vs. HGI diet (Table 2 and Figure 2A,B). As the glucose stimulus also varied, when we adjusted the total ISR to account for the difference in the incAUCglu, insulin secretion increased on the LGI diet and trended to decrease on the HGI diet compared to the Control diet, and was significantly increased on the LGI vs. HGI diet (Table 2). Insulin secretion was adjusted for insulin sensitivity in the statistical model.

### 3.4. Effect of Dietary Glycemic Index on Insulin Sensitivity, β-Cell Function and Insulin Clearance by Modeling

Insulin sensitivity by OGIS did not change with either diet intervention and there was no significant diet effect by GEE analysis. The calculated Matsuda index showed an increase in insulin sensitivity on the LGI diet, no change on the HGI diet, and a between diet difference by GEE (Table 2). The slope of the ISR dose response appeared steeper at higher glucose concentrations for both the LGI and HGI MTTs compared to the Control (Figure 2C,D), but glucose sensitivity, which represents the overall slope of this relationship, did not change or differ by diet intervention. Similarly, neither rate sensitivity nor the potentiation factor ratio changed (Table 2). The LGI arm was associated with an increase in total insulin clearance but did not change in the HGI arm, with a significant diet effect of LGI vs. HGI (Table 2). 

## 4. Discussion

Using a highly controlled feeding study, we compared the effects of a high vs. low GI diet in the setting of relative weight stability on insulin sensitivity and β-cell function, assessed during an MTT in adults with prediabetes. Glucose responses to the test meal were dependent on the GI of the meal, with lower glucoses and insulin secretion observed on the LGI diet. Although measures of β-cell function, including glucose sensitivity and rate sensitivity, did not change with the diet interventions, there was a relative increase in insulin secretion when adjusted for the lower overall glucose stimulus from the LGI meal test. Additionally, total insulin clearance increased in response to the LGI test meal.

Only one other controlled feeding study has assessed the impact of dietary GI on β-cell function in prediabetes. In that study 12 weeks of a low vs. high GI diet was combined with an exercise training and weight loss intervention in older, obese adults with prediabetes. Despite similar weight loss and improvements in insulin sensitivity, insulin secretion, adjusted for the change in glucose and insulin sensitivity during an OGTT, did not change in the low GI exercise group but increased in the high GI exercise group [16]. When examining changes in insulin secretion in response to a test meal, we observed relative increases in insulin secretion when adjusting for the lower glucose stimulus on the low GI diet and no change on the high GI diet. This could represent an early indication of improved β-cell responsiveness even after just 4 weeks on a low GI diet. However, other measures of β-cell function derived from the MTT did not change, and we did not observe any change in the disposition index, measured using an intravenous glucose tolerance test [18]. With a longer duration intervention, it is possible that reducing the demand for insulin secretion, such as with a low GI diet, could be beneficial for β-cell function but would need to be formally tested. Prospective observational studies found that primary hypersecretion was associated with a worse clinical and metabolic phenotype and predicted deterioration of glucose control over time [26]. A longer-term study that provided dietary advice to follow a high carbohydrate low GI vs. high GI diet in adults with IGT found that the low GI arm had an increase in the disposition index, as measured by an intravenous glucose tolerance test at 1 year, but no effect in the high GI arm [15].

The increase in total insulin clearance observed during the LGI MTT is interesting and may represent increased hepatic insulin extraction. Insulin clearance is strongly associated with insulin sensitivity [27,28,29,30], with this association specific for fractional extraction of insulin by the liver [27,28], but this cannot explain the increase in clearance here, as insulin sensitivity did not change with the LGI intervention diet. It is more likely that the lower insulin secretion during the LGI MTT resulted in greater insulin clearance, as insulin clearance is directly associated with the amount of insulin delivered to the liver, i.e., insulin secretion, as well as to the systemic circulation [27,30]. It also has been noted that hepatic insulin extraction can saturate at high rates of insulin secretion [27,28,30], and this could have been relieved by decreasing insulin delivery to the liver.

We found no effect of the GI diets on insulin sensitivity as determined by OGIS during the MTT, which is consistent with our results, estimated based on an intravenous glucose tolerance test [18]. In contrast to our findings, the OmniCarb study reported a reduction in the Matsuda index after 5 weeks on the low GI (GI 40), high carbohydrate diet, but no effect on a high GI (GI 65) diet in overweight adults without diabetes [17]. We calculated the Matsuda index for this study and found it increased on the LGI diet without changing on the HGI diet. This is the opposite from what was observed in OmniCarb. It is possible that the Matsuda index is not as accurate when used for data from a meal test, especially one that varied in glycemic index. Dietary advice to follow a high carbohydrate low GI vs. high GI diet in adults with IGT also failed to result in any change in insulin sensitivity after 1 year, but the differential in the GI between the two intervention arms in that study was quite small [15]. The lack of an independent effect of dietary GI on insulin sensitivity in prediabetes is also supported by the study comparing a low vs. high GI diet combined with exercise, which found both groups lost equal amounts of weight (−8.8%) and had similar improvements in insulin sensitivity as measured by hyperinsulinemic-euglycemic clamps [16].

The strengths of this study include the highly controlled feeding protocol where all the foods were provided to the participants, the large differential between the low and high GI diets (<35 vs. >70), the use of a baseline moderate Control diet, and the use of an MTT with modeling to assess β-cell function using a more physiologic stimulus. The study is limited by the small sample size, which prevents generalizability, and the short duration of the intervention, both factors affected by the cost of such studies and participant burden. It is important to note that diets include whole food, and it is impossible to isolate out one specific dietary component without altering other factors. In the case of this study, the LGI diet included higher fiber and fructose, while these were decreased on the HGI diet [18]. These other differences that accompany an LGI diet may have their own independent effects on glucose metabolism.

## 5. Conclusions

The data indicate that selecting foods with a low vs. high GI, at least in the short-term, does not have a major impact on insulin sensitivity or β-cell function. However, we cannot rule out the possibility that reducing the demand on the β-cell with a longer-term low GI diet could have a beneficial effect on β-cell function. The increase in insulin secretion during the MTT after adjusting for the lower glucose stimulus and improved insulin clearance could be an early indication. Longer intervention studies are needed to answer these questions.

## Figures and Tables

**Table 1:** Participant Baseline Characteristics.

	All N = 35	LGI N = 17	HGI N = 18	*p* Value
Age (years)	54.20 ± 1.57	58.06 ± 1.52	50.56 ± 2.44	0.01
Gender (M/F)	18/17	9/8	9/9	0.86
BMI (kg/m^2^)	32.44 ± 0.94	32.45 ± 1.16	32.42 ± 1.50	0.99
HbA1c (% and mmol/mol)	5.76 ± 0.06 39.48 ± 0.63	5.76 ± 0.06 39.44 ± 0.68	5.77 ± 0.10 39.53 ± 1.07	0.95
Screening fasting glucose (mmol/L)	5.82 ± 0.05	5.93 ± 0.06	5.73 ± 0.08	0.07
Screening 2 h glucose (mmol/L)	7.92 ± 0.22	7.71 ± 0.36	8.12 ± 0.27	0.36
IFG/IGT/IFG + IGT	14/5/16	9/2/6	5/4/10	0.31
Race (n)				0.38
White	24	13	11	
African American	5	1	4	
Asian	6	3	3	
Ethnicity (n)				0.95
Hispanic	4	2	2	
Non-Hispanic	31	15	16	

ANOVA for continuous variables and chi-square for categorical variables. Mean ± SEM.

**Table 2:** MTT Metabolic Outcomes by Diet Intervention.

	LGI Diet (*n* = 17)				HGI Diet (*n* = 18)				GEE Analysis LGI vs. HGI
	Control	LGI	Change	*p* Value	Control	HGI	Change	*p* Value	*p* Value
OGIS [mL/min·m^2^]	440.27 ± 12.06	446.26 ± 9.57	5.99 ± 2.49	0.37	407.63 ± 12.16	407.67 ± 11.25	0.04 ± 0.91	0.99	0.08
Matsuda Index	7.10 (5.39)	8.85 (6.25)	0.57 (2.62)	0.01	5.72 (3.60)	4.53 (3.22)	−0.24 (1.52)	0.41	0.001
Basal glucose [mmol/L]	4.62 ± 0.12	4.70 ± 0.11	0.08 ± 0.01	0.07	4.70 ± 0.11	4.65 ± 0.12	−0.05 ± 0.01	0.39	0.06
incAUCglu during MTT [mmol/L]	526.87 ± 49.72	393.31 ± 37.28	−133.56 ± 12.44	<0.001	532.06 ± 37.73	591.53 ± 38.05	59.47 ± 0.32	0.14	<0.001
Early Insulin Response (0–30 min) [pmol/mmol]	102.66 (55.3)	68.98 (59.73)	−24.25 (55.71)	0.02	166.96 (129.13)	107.55 (149.09)	−3.54 (60.3)	0.24	0.10 *
incAUCins/incAUCglu (0–240 min) [pmol/mmol]	85.46 (67.54)	103.03 (53.42)	−7.48 (47.44)	0.48	140.23 (262.29)	141.40 (111.53)	−7.18 (50.21)	0.13	0.50 *
Early C-peptide Response (0–30 min) [nmol/mmol]	0.41 (0.25)	0.33 (0.24)	−0.008 (0.03)	0.75	0.48 (0.26)	0.49 (0.33)	−0.005 (0.03)	0.13	0.28 *
incAUCcp/incAUCglu (0–240 min) [nmol/mmol]	0.71 (0.28)	0.78 (0.38)	0.06 (0.32)	0.03	0.88 (0.49)	0.81 (0.48)	−0.08 (0.24))	0.03	0.003 *
Glucose Sensitivity [pmol/min·m^2^·mM]	65.23 (48.56)	91.23 (26.98)	−6.85 (51.91)	0.69	95.75(50.04)	106.52 (75.55)	7.59 (35.06)	0.13	0.29
Rate Sensitivity [pmol/m^2^·mM]	1017.97 (963.20)	813.77 (632.38)	−287.73 (639.05)	0.82	898.35 (987.52)	916.39 (1165.96)	32.73 (793.58)	0.65	0.35
Potentiation Factor Ratio	1.18 (0.65)	1.08 (0.45)	−0.20 (0.56)	0.13	1.165 (0.30)	1.28 (0.48)	0.02 (0.49)	0.84	0.25
ISR @ 5mmol glucose [pmol/min·m^2^]	135.85 ± 12.13	134.54 ± 10.54	−1.31 ± 1.59	0.82	171.46 ± 13.30	148.85 ± 15.95	−22.61 ± 2.65	0.02	0.20 *
Total Insulin Secretion [nmol/m^2^]	70.18 (32.70)	60.45 (29.17)	−8.57 (19.50)	0.02	82.25 (435.67)	82.23 (36.64)	−1.88 (12.03)	0.87	0.002 *
Total IS/incAUCglu [nmol·L/mmol·m^2^]	162.27 (71.89)	189.10 (100.29)	29.00 (52.25)	0.002	170.49 (114.65)	151.51 (80.38)	−11.48 (38.50)	0.10	0.001 *
Basal Insulin Clearance [pmol/L]	1.86 (1.12)	2.21 (0.76)	−0.063 (0.51)	0.24	1.73 (0.59)	1.65 (1.09)	−0.11(0.67)	0.99	0.314
Total Insulin Clearance [pmol/L]	1.37 ± 0.093	1.56 ± 0.09	0.19 ± 0.001	0.01	1.11 ± 0.09	1.07 ± 0.09	−0.04 ± 0.002	0.21	<0.001

Mean ± SEM for normally distributed variables and median (IQR) for non-normally distributed variables. Paired *t*-test analysis for within treatment arm comparison. GEE analysis for comparison of LGI vs. HGI response to the intervention adjusted for age and baseline. * GEE also adjusted for OGIS.

## Data Availability

Deidentified data supporting this manuscript will be made available upon reasonable request.

## Supplementary Materials

The following supporting information can be downloaded at: https://www.mdpi.com/article/10.3390/nu14040887/s1, Table S1. Meal Test Composition by Diet; Figure S1. Consort Flow Diagram.
