# Supplementary material for: Effect of Dietary Glycemic Index on β-Cell Function in Prediabetes: A Randomized Controlled Feeding Study
Source: Nutrients. 2022 Feb 19;14(4):887. doi: 10.3390/nu14040887 (PMC8877348; doi:10.3390/nu14040887)
Supplement: Supplementary file 1 [file nutrients-14-00887-s001.zip › nutrients-1574667-supplementary.pdf]

Figure S1: CONSORT Flow Diagram

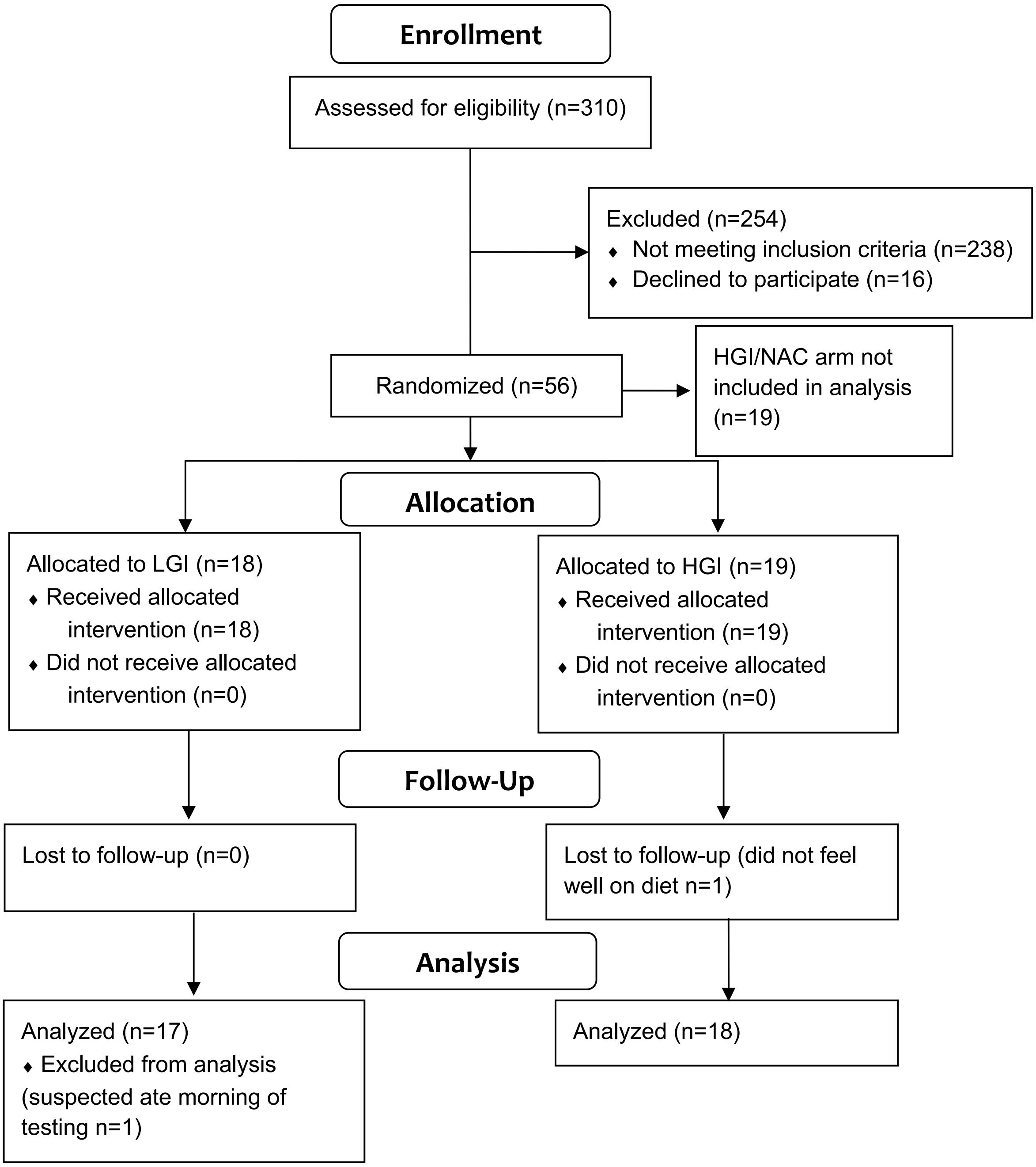

Supplemental Table S1. Meal Test Composition by Diet

Control Diet							
**Food Description**	**Grams**	**Energy (kcal)**	**Protein (%)**	**Fat (%)**	**Carb (%)**	**Fiber, total dietary**	**Glycemic Index**
Rice, brown, long-grain, cooked	240	266.4	9.36	7.35	83.29	4.32	55
DGI Chili base (Sat. night)	120	37.2	11.4	26.77	61.84	1.45	23
Beef, ground, 90%10% fat, crumbles, cooked	46	105.8	51.22	48.78	0	0	-
Oil, canola	9	79.56	0	100	0	0	-
Orange juice, unsweetened	125	56.25	5.86	1.16	92.97	0.25	50
**Meal Totals**	**540**	**545.21**	**15.57**	**29.61**	**54.82**	**6.02**	
**LGI Diet**							
**Food Description**	**Grams**	**Energy (kcal)**	**Protein (%)**	**Fat (%)**	**Carb (%)**	**Fiber, total dietary**	**Glycemic Index**
Beans, kidney, cooked	120	152.4	26.6	3.45	69.95	7.68	23
DGI Chili base (Sat. night)	120	37.2	11.4	26.77	61.84	1.45	23
Tortillas, flour	52	162.24	10.76	22.62	66.62	1.61	30
Beef, ground, 90%10% fat, crumbles, cooked	20	46	51.22	48.78	0	0	-
Butter, salted	1	7.17	0.46	99.5	0.03	0	-
Oil, canola	9	79.56	0	100	0	0	-
Apple juice,	130	59.8	0.86	2.5	96.64	0.26	40

unsweetened							
**Meal Totals**	**452**	**544.37**	**15.74**	**29.76**	**54.49**	**11.01**	
**HGI Diet**							
**Food Description**	**Grams**	**Energy (kcal)**	**Protein (%)**	**Fat (%)**	**Carb (%)**	**Fiber, total dietary**	**Glycemic Index**
Rice, white, long-grain, regular, cooked, unenriched, with salt	140	182	8.54	2	89.46	0.56	109
DGI Chili base (Sat. night)	100	31	11.4	26.77	61.84	1.21	23
Tortillas, corn	28	61.04	10.04	11.3	78.66	1.76	52
Beef, ground, 90%10% fat, crumbles, cooked	51	117.3	51.22	48.78	0	0	-
Oil, canola	9.5	83.98	0	100	0	0	-
Cranberry juice cocktail, bottled	132	71.28	0	1.64	98.36	0	68
**Meal Totals**	**460.5**	**546.6**	**15.28**	**29.66**	**55.06**	**3.54**	
